# Geriatric Nutritional Risk Index as a Prognostic Marker for Patients With Metastatic Castration-Resistant Prostate Cancer Receiving Docetaxel

**DOI:** 10.3389/fphar.2020.601513

**Published:** 2021-01-25

**Authors:** Li-Wen Chang, Sheng-Chun Hung, Jian-Ri Li, Kun-Yuan Chiu, Cheng-Kuang Yang, Chuan-Shu Chen, Kevin Lu, Cheng-Che Chen, Shu-Chi Wang, Chia-Yen Lin, Chen-Li Cheng, Yen-Chuan Ou, Shun-Fa Yang, Chiann-Yi Hsu, Szu-Hang Ho, Shian-Shiang Wang

**Affiliations:** ^1^ Division of Urology, Department of Surgery, Taichung Veterans General Hospital, Taichung, Taiwan; ^2^ Institute of Medicine, Chung Shan Medical University, Taichung, Taiwan; ^3^ Department of Medicine and Nursing, Hungkuang University, Taichung, Taiwan; ^4^ Department of Applied Chemistry, National Chi Nan University, Nantou, Taiwan; ^5^ School of Medicine, National Yang Ming University, Taipei, Taiwan; ^6^ Department of Medical Research, Taichung Veterans General Hospital, Taichung, Taiwan; ^7^ Department of Urology, Tungs’ Taichung Metro Harbor Hospital, Taichung, Taiwan; ^8^ Biostatistics Task Force of Taichung Veterans General Hospital, Taichung, Taiwan

**Keywords:** geriatric nutritional risk index, metastasis castration resistant prostate cancer, chemotherapy, DOCETAXEL, survival

## Abstract

**Purpose:** To investigate the prognostic efficacy of the Geriatric Nutritional Risk Index (GNRI) in patients with metastatic Castration–resistant Prostate Cancer (mCRPC) receiving docetaxel as the first line of treatment.

**Methods:** We retrospectively reviewed patients with mCRPC and receiving first line docetaxel in Taichung Veterans General Hospital from 2006 to 2012. The GNRI was calculated using serum albumin and body mass index, with a poor nutritional status defined as GNRI <92.0. Multivariate Cox-regression analysis was used to evaluate the risk of survival.

**Results:** One-hundred seventy patients with mCRPC were included. One-hundred twenty-five patients were of normal nutritional status (GNRI ≥92) and 45 patients were of poor nutritional status (GNRI <92). The cumulative docetaxel dosage was 600 (360–1,185) mg in the normal nutritional status group and 360 (127.5–660) mg in the poor nutritional status group (*p* < 0.001). The median overall survival from mCRPC was 30.39 months in the good nutritional status group and 11.07 months in the poor nutritional status group (*p* of log rank <0.001). In a multivariate model, poor nutritional status was an independent risk factor in overall survival (Hazard Ratio [HR] = 5.37, 95% Confidence Interval [CI] 3.27–8.83), together with a high metastatic volume (HR = 4.03, 95% CI 2.16–7.53) and docetaxel cumulative dosage (HR = 0.999, 95% CI 0.999–0.9998).

**Conclusion:** Poor nutritional status with a GNRI <92 is associated with shorter progression free survival and overall survival in mCRPC patients treated with docetaxel. Metastatic volume and cumulative docetaxel dosage are also independent prognostic factors in overall survival.

## Introduction

Prostate cancer accounts for the most common type of cancer in men, while having the second highest cancer related death rate (Siegel et al., 2018). Although the incidence of metastatic prostate cancer has decreased since recommendations for Prostate-specific Antigen (PSA) exams have increased, it has nevertheless still increased by >2.7% per year since 2012, with the annual burden expected to increase 42% by the year 2025 (Kelly et al., 2018). Due to the rise in prostate cancer and its progress depending upon the androgen signal pathway, androgen deprivation therapy with medical or surgical castration has been used as an effective therapeutic strategy since 1942 (Huggins, 1942). Nevertheless, disease progression occurs despite under castration level as the condition of metastatic Castration-resistant Prostate Cancer (mCRPC) remains the leading cause of death in prostate cancer patients (Hussain et al., 2009).

In 2004, chemotherapy with docetaxel was approved as the standard form of treatment for mCRPC according to the SWOG 99–16 study and the TAX-327 study (Petrylak et al., 2004; Tannock et al., 2004). Three weekly doses of Docetaxel 75 mg/m^2^, along with androgen deprivation therapy improved mCRPC, with a median 18.9 months overall survival period, a 45 percent PSA response rate and a 35 percent improved symptom rate (Tannock et al., 2004). Clinical characteristics including pretreatment PSA, Alkaline phosphatase (ALP), Lactate dehydrogenase (LDH), performance status, hemoglobin, Gleason sum, visceral or liver metastases, PSA doubling time, clinical significance of pain and number of metastases have all been recognized as the prognostic predictive factors for Overall Survival (OS) in mCRPC patients treated with docetaxel (Halabi et al., 2003; Armstrong et al., 2007; Armstrong et al., 2010).

Nutritional status has been reported as being significantly associated with the clinical outcome of many human pathologic conditions, particularly in malignant diseases (Argilés, 2005). The use of the Geriatric Nutritional Risk Index (GNRI), which consists of serum albumin levels and the ratio of actual and ideal body weights, is a simple and available nutritional assessment initially designed to predict survival in elderly hospitalized patients (Bouillanne et al., 2005). Meta-analysis, which conducts the outcome of many human malignancies, has reported that a low GNRI is significantly associated with poor overall survival and cancer specific survival (Lv et al., 2019). Okamoto et al. further reported that a low GNRI can predict a higher mortality risk in patients with metastatic Hormone Sensitive Prostate Cancer (mHSPC) (Okamoto et al., 2019). However, there has been no study discussing the association between GNRI and mCRPC. Therefore, the aim of this study was to retrospectively investigate the impact of GNRI on the survival outcome of mCRPC patients treated with docetaxel.

## Patients and Methods

The current study enrolled patients diagnosed with mCRPC at Taichung Veterans General Hospital from 2006 to 2012. All patients are in accordance with the pathology proved metastatic prostate adenocarcinoma and clinical stage 4 disease (AJCC, American Joint Committee on Cancer prostate cancer staging). Patients received informed consent forms according to the certifications of the Institute Review Board of Taichung Veterans General Hospital, CE20173B. Inclusion criteria for mCRPC were pathology confirmed prostate adenocarcinoma and progression under the castration level (testosterone level <50 ng/dl).

Androgen deprivation therapy (involving either surgical castration through orchiectomy or medical castration involving LH-RH agonists or antagonists) began from metastasis prostate cancer being diagnosed to throughout the entire period of mCRPC. The protocol for chemotherapy with docetaxel was 75 mg/m^2^ during a 3-weeks interval, in combination with 10 mg prednisone daily, while 50 mg/m^2^ over a 2-weeks interval could be prescribed instead, and later transferred into standard 3-weeks cycle counts. PSA progression was defined according to the Prostate Cancer Working Group second publication (PCWG2) criteria (Scher et al., 2008).

The nutritional status was evaluated according to previous reports, with GNRI values being calculated as 1.489 × serum albumin level (g/L) + 41.7 × [actual body weight (kg)/ideal body weight (kg)] (Yamada et al., 2008). The ideal body weight was identified as [height (m)]^2^ × 22 (kg/m^2^). The value of the actual body weight/ideal body weight was set to one when the actual body weight exceeded the ideal body weight. Poor nutritional status was defined as a GNRI <92.0, based upon previous literature (Gu et al., 2015; Bo et al., 2016). Accordingly, patients were divided into either a normal nutrition group (GNRI ≥92.0) or poor nutrition group (GNRI <92.0).

Patient characteristics which were recorded included age at mCRPC, Eastern Cooperative Oncology Group (ECOG) performance status, PSA at initial metastatic prostate cancer diagnosis, nadir PSA at mHSPC, hormone sensitive duration (in months, as defined from initial ADT to mCRPC), Gleason score (G/S), hypertension, diabetes mellitus, coronary artery disease, Body Mass Index (BMI, kg/m^2^) and metastatic status (bone, lymph node, lung, liver and brain).

Metastatic status at mCRPC was defined according to previous clinical trials. High volume disease was defined as the presence of visceral metastases or four or more bone lesions, with more than one lesion being located beyond the vertebral bodies and pelvis, when compared to low volume disease (Sweeney et al., 2015). High-risk disease was defined as a patient having any two of the following: 1) three or more bone metastases as seen on a bone scan, 2) a Gleason sum ≥8, and 3) any visceral metastases (Fizazi et al., 2017).

End point evaluated the Overall Survival (OS) from mCRPC and PSA Progression-free Survival (PFS) using the Kaplain-Meier survival curve and log-rank test. The Mann–Whitney *U* test was used for continuous variables and expressed as medians [Interquartile Ranges (IQRs)]. The Fisher’s exact *t*-test was used for categorical variables and expressed as percentages. Univariate and multivariate Cox hazard regression were used to estimate the Hazard Ratio (HR) and 95% Confidence Interval (CI) for the association between the variables and OS. Analyses were performed using SAS software version 9.2 (SAS Institute, Inc, Cary, NC, United States). A *p*-value of <0.05 was considered statistically significant.

## Results

One-hundred twenty-five patients categorized as normal nutrition (GNRI ≥92.0) and 45 patients grouped as poor nutrition (GNRI <92.0) were enrolled in the study. Table 1 shows the patient characteristics for those enrolled in the study. The median age was 74.09 (IQR 67.73–80.14) in the normal nutrition group and 78.77 (72.38–81.54) in the poor nutrition group. During a median follow up period of 22.49 (11.35–41.32) months, 107 of 170 patients died of mCRPC after the first line of docetaxel. Significant between-group differences included age at mCRPC (*p* = 0.023*), ECOG performance state (*p* < 0.001**), BMI (*p* < 0.001**), LDH (*p* = 0.025*), ALP (*p* = 0.002*), high risk metastases status (*p* = 0.001**) and high volume metastases (*p* = 0.001**). The incidence of lung metastases and visceral metastases were lower in the normal nutrition group when compared to the poor nutrition group (9.60 vs. 26.67%, *p* = 0.010* and 16.80 vs. 42.22%, *p* = 0.001**, respectively). Cumulative dosage of docetaxel for mCRPC was 600 (360–1,185) mg in the normal nutrition group, while it was 360 (127.5–660) mg in the poor nutrition group (*p* < 0.001**). After docetaxel treatment, PSA decline was −55.11 (−82.02–6.95) % in the normal nutrition group, compared to −36.54 (−57.82–20.97) % in the poor nutrition group (*p* = 0.042*).

**TABLE 1:** Baseline characteristics of patients with mCRPC.

	GNRI≥92 (n = 125)		GNRI<92 (n = 45)		*p* value
Age at mCRPC	74.09	(67.73–80.14)	78.77	(72.38–81.54)	0.023*
Performance state (n = 169)					<0.001**
0	54	(43.20%)	8	(18.18%)	
1	60	(48.00%)	21	(47.73%)	
2	11	(8.80%)	15	(34.09%)	
PSA at initial	126.00	(37.51–400.5)	97.81	(22.95–400)	0.260
Nadir PSA at mHSPC	0.42	(0.11–2.02)	0.77	(0.17–3.04)	0.339
Hormon sensitive period	32.96	(15.66–75.22)	26.64	(15.61–51.32)	0.242
Gleason score	9.00	(7–9)	8.00	(7–9)	0.669
Hypertension	42	(33.60%)	17	(37.78%)	0.747
Diabetes mellitus	14	(11.20%)	6	(13.33%)	0.912
Coronary artery disease	14	(11.20%)	7	(15.56%)	0.619
BMI	24.65	(22.87–26.97)	23.10	(20.24–25.05)	0.001**
PSA at mCRPC	21.90	(9.82–60)	28.70	(10.49–118)	0.294
LDH	228.00	(189.25–270.75)	268.00	(211–364)	0.025*
ALP	113.00	(77–187.75)	149.00	(102.5–658.5)	0.002**
High risk metastases	48	(38.40%)	31	(68.89%)	0.001**
High volume metastases (at CRPC)	66	(52.80%)	37	(82.22%)	0.001**
Metastases site					
Bone metastases^a^	123	(98.40%)	45	(100%)	1.000
Lymph node metastases	69	(55.20%)	27	(60.00%)	0.703
Lung metastases	12	(9.60%)	12	(26.67%)	0.010*
Liver metastases^a^	12	(9.60%)	3	(6.67%)	0.762
Brain metastases	2	(1.60%)	4	(8.89%)	0.072
Visceral metastases	21	(16.80%)	19	(42.22%)	0.001**
Docetaxel					
Docetaxel 75 mg/m^2^ courses (n = 112)	5.00	(3–9)	3.00	(1–5)	<0.001**
Docetaxel 60 mg/m^2^ courses (n = 26)	3.50	(2.25–10.75)	6.00	(6–7)	0.186
Docetaxel 50 mg/m^2^ courses (n = 38)	8.00	(5.5–15)	5.00	(2.5–11)	0.108
Cumulative docetaxel dosage (mg)	600.00	(360–1,185)	360.00	(127.5–660)	<0.001**
PSA before treatment	38.03	(13.43–200)	99.02	(14.1–342)	0.321
Nadir PSA after treatment	16.00	(3.58–90.76)	38.75	(4.55–160.25)	0.294
PSA decline (%)	−55.11	(−82.02–6.95)	−36.54	(−57.72–20.97)	0.042*
Subsequent abiraterone acetate	54	(43.20%)	9	(20.00%)	0.010*
Subsequent enzalutamide	17	(13.60%)	3	(6.67%)	0.333
Median follow up time from mCRPC	30.39	(16.7–45.02)	11.07	(7.38–16.14)	<0.001**

Chi-Square test.

^a^ Fisher's Exact test. Mann-Whitney test. *p< 0.05, **p< 0.01.

Continuous data were expressed as a median (IQR).

Categorical data were expressed as a number and percentage.

GNRI, Geriatric Nutritional Risk Index; mCRPC, metastatic Castration–resistant Prostate Cancer; PSA, Prostate specific antigen; mHSPC, metastatic hormone sensitive prostate cancer; BMI, body mass index; LDH, lactate dehydrogenase; ALP, alkaline phosphatase.

**FIGURE 1:**
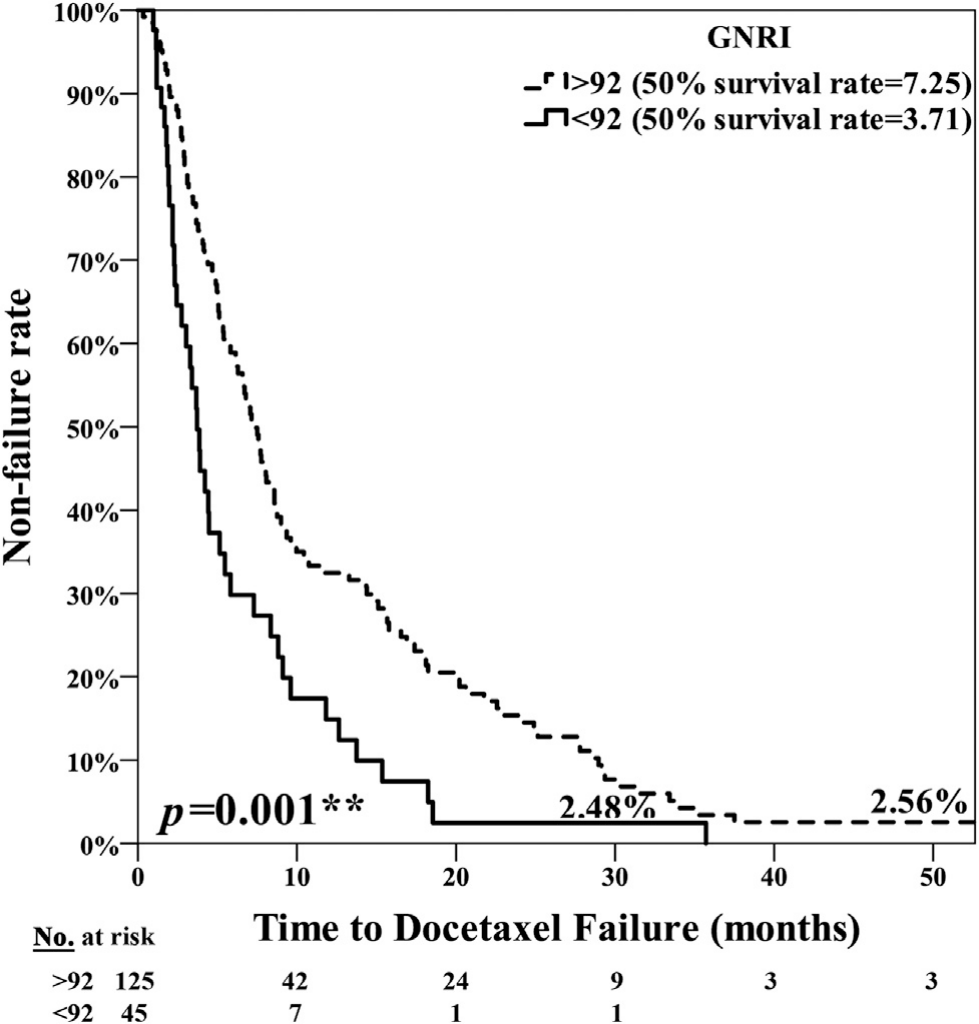
Kaplan-Meier curve for PSA Progression Free Survival (PFS) among the normal nutrition group (GNRI ≥92.0) and poor nutrition group (GNRI <92.0), median 7.25 months vs. 3.71 months, respectively (*p* = 0.001**).

**FIGURE 2:**
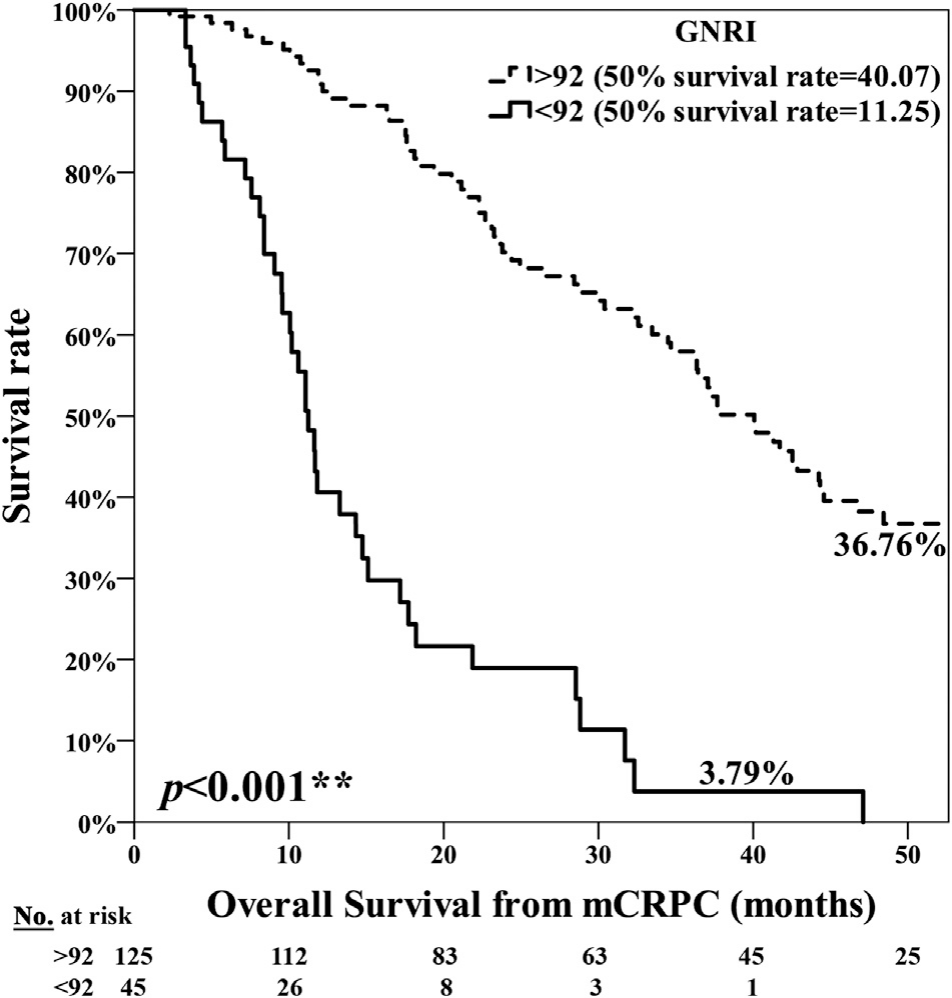
Kaplan-Meier curve for Overall Survival (OS) among the normal nutrition group (GNRI ≥92.0) and poor nutrition group (GNRI <92.0), median 40.07 months vs. 11.25 months, respectively (*p* < 0.001**).

**FIGURE 3:**
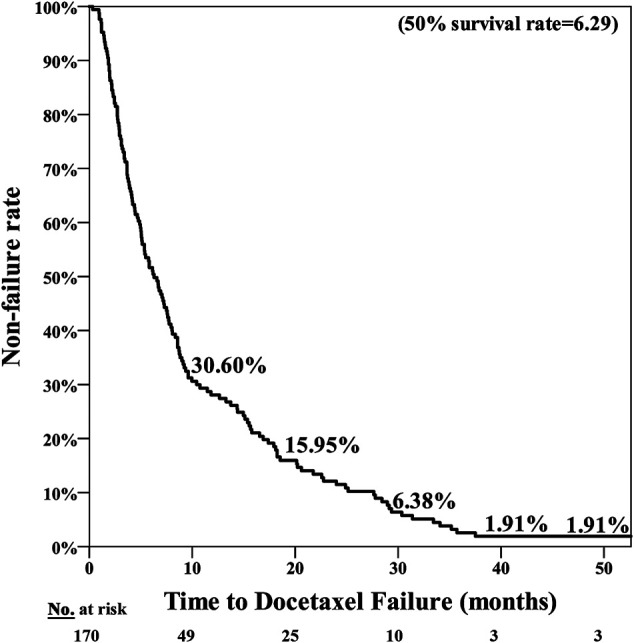
PSA Progression Free Survival (PFS) for mCRPC patients treated with docetaxel for a median of 6.29 months.

**FIGURE 4:**
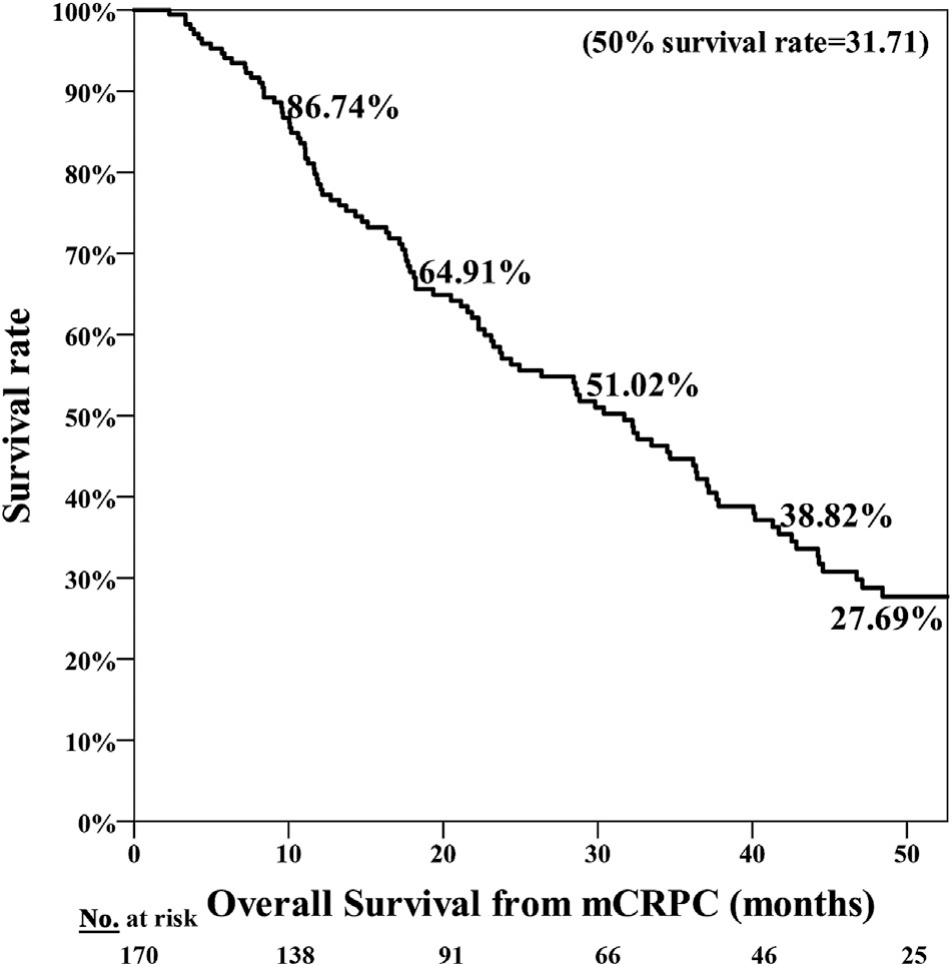
Overall Survival (OS) for mCRPC patients treated with docetaxel for a median 31.71 months.

The poor nutrition group exhibited a significantly poorer prognosis, with the median period for PFS being 7.25 months in the normal nutrition group and 3.71 months in the poor nutrition group (*p* = 0.001**) (Figure 1). The median period for OS was 40.07 months in the normal nutrition group and 11.25 months in the poor nutrition group (*p* < 0.001**) (Figure 2). Among all the mCRPC patients treated with docetaxel, the median period for PFS was 6.29 months (Figure 3), and for OS 31.71 months (Figure 4).

Table 2 demonstrates the results of univariate and multivariate Cox hazard regression analyses for overall survival from mCRPC. After adjustment, high metastases volume (HR = 4.03, 95% CI = 2.16–7.53, *p* < 0.001**), cumulative docetaxel dosage (HR = 0.999, 95% CI = 0.999–0.9998, *p* = 0.002**) and a GNRI<92 (HR = 5.37, 95% CI = 3.27–8.83, *p* < 0.001**) were all independent risk factors affecting overall survival from mCRPC.

**TABLE 2:** Uni-Multi variant analysis for overall survival.

	Univariate			Multivariate			
	HR	95% CI	*p* value	HR	95% CI	*p* value
Age at mCRPC	1.01	(0.99–1.04)	0.254	0.98	(0.95–1.00)	0.071
Performance state							
0	Ref					
1	1.30	(0.84–1.99)	0.237			
2	2.48	(1.42–4.35)	0.001**			
PSA at initial	1.00	(1.00–1.00)	0.804			
Nadir PSA at mHSPC	1.00	(1.00–1.00)	0.079			
Hormone sensitive period	0.99	(0.99–0.999)	0.026*	1.00	(0.99–1.00)	0.706
Gleason score	1.00	(0.85–1.18)	0.998			
BMI	0.94	(0.89–0.99)	0.027*	0.99	(0.93–1.05)	0.656
PSA at mCRPC	1.00	(1.00–1.00)	0.095			
LDH	1.004	(1.002–1.01)	<0.001**			
ALP	1.001	(1.0005–1.001)	<0.001**			
High risk metastases	3.06	(2.04–4.59)	<0.001**	0.82	(0.48–1.40)	0.468
High volume metastases (at CRPC)	4.61	(2.93–7.25)	<0.001**	4.03	(2.16–7.53)	<0.001**
Docetaxel							
Docetaxel 75 mg/m^2^ courses	0.95	(0.91–0.99)	0.008**			
Docetaxel 60 mg/m^2^ courses	0.84	(0.73–0.97)	0.016*			
Docetaxel 50 mg/m^2^ courses	1.01	(0.95–1.06)	0.830			
Cumulative docetaxel dosage	0.999	(0.999–0.9997)	<0.001**	0.999	(0.999–0.9998)	0.002**
PSA before treatment	1.0003	(1.0001–1.0005)	0.008**	1.00	(1.00–1.00)	0.230
Nadir PSA after treatment	1.00	(1.00–1.00)	0.146			
GNRI							
≥92	Ref			Ref		
<92	5.64	(3.64–8.73)	<0.001**	5.37	(3.27–8.83)	<0.001**

Cox regression. *p< 0.05, **p< 0.01.

GNRI, Geriatric Nutritional Risk Index; mCRPC, metastatic Castration–resistant Prostate Cancer; PSA, Prostate specific antigen; mHSPC, metastatic hormone sensitive prostate cancer; BMI, body mass index; LDH, lactate dehydrogenase; ALP, alkaline phosphatase.

The adverse events are shown in Table 3. Incidences of febrile neutropenia (35.56 vs. 12.00%, *p* = 0.001**) and liver function impairment with elevated AST (Aspartate aminotransferase)/ALT (Alanine aminotransferase) (17.78 vs. 4.00%, *p* = 0.006**) were higher in the poor nutrition group.

**TABLE 3:** Adverse events.

	GNRI<92 (n = 45)		GNRI>92 (n = 125)		*p* value
	n	%	n	%	
Neutropenia					0.028*
Grade 1/2	4	(8.89%)	30	(24.00%)	
Grade 3/4	15	(33.33%)	48	(38.40%)	
Febrile neutropenia					0.001**
Grade 3/4	16	(35.56%)	15	(12.00%)	
Rash^a^					0.683
Grade 1/2	1	(2.22%)	7	(5.60%)	
Fatigue					0.086
Grade 1/2	11	(24.44%)	14	(11.20%)	
Grade 3/4	0	(0.00%)	1	(0.80%)	
Elevated AST/ALT^a^					0.006**
Grade 1/2	8	(17.78%)	5	(4.00%)	
Diarrhea^a^					0.286
Grade 1/2	2	(4.44%)	2	(1.60%)	
Nausea					0.247
Grade 1/2	6	(13.33%)	17	(13.60%)	
Grade 3/4	1	(2.22%)	0	(0.00%)	

Chi-Square test.

^a^ Fisher's Exact test. *p< 0.05, **p< 0.01.

GNRI, Geriatric Nutritional Risk Index; AST, Aspartate aminotransferase; ALT, Alanine aminotransferase.

## Discussion

Our results suggest that nutritional status evaluation using the GNRI is a useful tool in predicting survival in mCRPC patients treated with docetaxel. To the best of our knowledge, this was the first study to have shown the positive correlation between the GNRI and mCRPC. Additionally, a high metastases volume and one’s cumulative docetaxel dosage were independent risk factors associated with overall survival in mCRPC patients.

Poor nutritional status is a common problem in elderly patients with advanced disease, and is associated with decreased protein reserves and a negative protein and energy balance which may lead to cachexia status and mortality (Dent et al., 2015). Cachexia may be characterized by loss of muscle, with or without the loss of fat mass, which in turn leads to weight loss. This occurs in the majority of cancer patients prior to death and is responsible for the deaths of 22% of cancer patients (Argilés et al., 2010). Malnutrition and cachexia also suppress the synthesis of serum albumin in advanced cancer patients as a result of tumor progression, the immune response to the tumor, and anticancer therapies (Gupta and Lis, 2010). GNRI is one of the more simplified and convenient predictive tools and consists of BMI and the albumin level of each patient. This is associated with an elevated risk of all cause mortality in many human diseases such as diabetes mellitus, cardiovascular disease, end stage renal disease and various cancers (Hao et al., 2019). Lidoriki et al. concluded that low GNRI scores were associated with an increased risk for both developing postoperative complications and impaired survival in cancer patients, as seen by a systemic review of eighteen studies associated with GNRI and cancers (Lidoriki et al., 2020). Lee et al. conducted GNRI studies from a randomized controlled trial of extended-stage disease small cell lung cancer treated with etoposide plus cisplatin, finding that a low GNRI value was associated with systemic inflammation, nutritional status and a poor prognosis outcome (Lee et al., 2020). Similar results were also found that GNRI in elderly gastric cancer patients treated with a gastrectomy as a simple, cost-effective, and promising nutritional index (Hirahara et al., 2020).

Although prostate cancer is a relatively slow-growing disease, it may progress aggressively if metastases tumors exist, particularly in those patients resistant to castration therapy in regards to mCRPC (Petrylak et al., 2004; Tannock et al., 2004). Previous studies have shown the association between nutritional status and oncology outcomes of metastases prostate cancer patients. Montgomery et al. evaluated the effect of BMI on outcomes of metastatic prostate cancer patients as taken from phase III randomized studies coordinated by the Southwest Oncology Group. The team found that a higher BMI was associated with better overall and progression-free survival in patients with androgen dependent metastatic prostate cancer (Montgomery et al., 2007). Wang et al. investigated the pretreatment serum albumin/globulin ratio as being an independent prognostic biomarker for progression free survival and cancer specific survival in patients with metastatic prostate cancer who had been receiving maximal androgen blockade treatment (Wang et al., 2018).

Application of the GNRI for mHSPC was also reported and concluded to be an independent risk factor surrounding cancer-specific survival with a hazard ratio of 1.76. Additionally, the study group designed a risk score comprised of a GNRI <92.0, Hb < 13.0 g/dl, and LDH >222 IU/L as being effective in predicting survival (Okamoto et al., 2019). The difference in our study is that it was specifically focused on mCRPC, which is the most lethal stage of prostate cancer with a median survival period of 17.5–18.9 months, where our results revealed the strong association between GNRI and overall survival with a hazard ratio of 5.37 (Petrylak et al., 2004; Tannock et al., 2004). Furthermore, our results also revealed that patients with a poor nutritional status had a median survival period of 11.25 months, compared to 40.07 months in patients with a normal nutritional status. When a tumor progressed and became refractory to castration therapies, chemotherapy may then become one of the most effective means of management. Patients experiencing malnutrition and weight loss as the result of advanced tumor progression and associated cachexia appeared to develop more toxicity and intolerance to chemotherapies, which in turn caused poorer outcomes (Andreyev et al., 1998).

As a nutrition-related risk index, GNRI is different from BMI in increasing the weighting of albumin rather than body weight, thus to differentiate malnutrition patients with high BMI and well nutrition patients with low BMI (Bouillanne et al., 2005). Our result also supports this finding that even adjusted with BMI, low GNRI associated with 5.37-fold increase risk of death in multivariat analysis in Table 2. Additionally, BMI alone did not have any prognosis value after adjust in renal cell carcinoma, non-small cell lung cancer, small cell lung cancer and gastric cancer (Miyake et al., 2017; Shoji et al., 2018; Hirahara et al., 2020; Lee et al., 2020).

Ever since the results of the TAX 327 and SWOG 99–16 studies were published in 2004, Docetaxel became the standard form of treatment in mCRPC patients for more than 10 years, until the reports surrounding abiraterone acetate and enzalutamide were published (Petrylak et al., 2004; Tannock et al., 2004; de Bono et al., 2011; Scher et al., 2012). Both improvements in survival and reduction in bone pain are the advantages of chemotherapy, although grade 3 or four neutropenic fevers, nausea and vomiting, as well as cardiovascular events limit its application in geriatric patients suffering from poor nutrition. The efficacy of docetaxel in healthy senior adults appears to be compatible with younger patients as well, however its tolerance in frail senior adults has not been well studied (Droz and Chaladaj, 2008). Our results also revealed a higher incidence of complications, including febrile neutropenia and liver function impairment in the poor nutrition group. As one option for deciding upon aggressive treatment in patients diagnosed with advanced disease, the GNRI may be a useful tool in making both the decision and choice for which drugs should be prescribed.

There were also limitations in our study. The retrospective design and unplanned subset analysis restricted the power to determine the prognostic role of GNRI index and prospective cohort study is needed to overcome the potential bias. There is also lack of relative animal model study. Additionally, since the advent of new androgen target agents in 2014, the impact of abiraterone acetate and enzalutamide has not been well characterized, leading to information bias. A higher proportion of patients in the normal nutrition group received abiraterone acetate, and this may be due to the better tolerability for subsequent therapies in well nutrition patients. Further large-scale prospective studies need to be conducted in order to better explore the optimal treatment for mCRPC patients, particularly when it involves androgen receptor axis-targeted agents.

## Conclusion

Poor nutrition status with a GNRI <92 is associated with less progression free survival and overall survival in mCRPC patients treated with docetaxel. Metastatic volume and cumulative docetaxel dosage in patients are also independent prognostic factors surrounding overall survival.

## Ethics Statement

The studies involving human participants were reviewed and approved by certification at Taichung Veteran General Hospital, Taiwan, with Certification of approval with IRB: CE20173B. The patients/participants provided their written informed consent to participate in this study.

## Author Contributions

Study design and protocol development: L-WC, S-CH and S-SW. Manuscript writing and editing: L-WC. Statistical analysis: S-CH and C-YH. Data collection and patient management: L-WC, S-CH, J-RL, K-YC, C-KY, C-SC, KL, C-CC, S-CW, C-YL, C-LC, Y-CO, S-FY, S-HH, and S-SW. Supervision and revision: S-SW and K-YC.

## Conflict of Interest

The authors declare that the research was conducted in the absence of any commercial or financial relationships that could be construed as a potential conflict of interest.
